# Beyond colonization: *Candida albicans* exhibits substantial pathogenic potential in cystic fibrosis environments

**DOI:** 10.1038/s41522-025-00889-2

**Published:** 2025-12-19

**Authors:** Natasa Radakovic, Nikola Plackic, Jelena Djuris, Joachim Morschhäuser, Aleksandar Sovtic, Mihail Basa, Predrag Minic, Fabio Zobi, Aleksandar Pavic

**Affiliations:** 1 Institute of Molecular Genetics and Genetic Engineering, University of Belgrade, Belgrade, Serbia; 2 University of Belgrade - Faculty of Pharmacy, Belgrade, Serbia; 3 Institute of Molecular Infection Biology, University of Würzburg, Würzburg, Germany; 4 Mother and Child Health Institute of Serbia “Dr. Vukan Čupić”, Belgrade, Serbia; 5 Faculty of Medicine, University of Belgrade, Belgrade, Serbia; 6 Department of Chemistry, Fribourg University, Fribourg, Switzerland

**Keywords:** Microbiology, Biofilms, Environmental microbiology, Pathogens

## Abstract

Cystic fibrosis (CF) sputum represents a highly permissive niche for microbial colonization, yet the contribution of *Candida albicans* to disease progression remains insufficiently investigated despite its frequent detection in CF airways. We hypothesized that the heterogeneous nature of CF lung, reflected through the emergence of oxygen-depleted niches during disease progression, modulates *C. albicans* pathogenicity and antifungal susceptibility. Using complementary in vitro and in vivo approaches, we show that clinical CF isolates of *C. albicans* are virulent in CF-mimicking environments. Synthetic CF medium (SCFM2) supported robust filamentation, with oxygen-nutrient interplay critically shaping fungal growth and drug response. In a novel CF infection model using zebrafish morphants, we observed heightened susceptibility to *C. albicans* compared to wild-type embryos. Reporter strains showed elevated *ECE1* expression, indicating increased candidalysin production and virulence in vivo. Our study provides compelling evidence that CF isolates of *C. albicans* have pathogenic potential, warranting consideration in future therapeutic strategies.

## Introduction

The lung microbiota has a profound impact on clinical outcomes in chronic respiratory diseases such as asthma, bronchiectasis, chronic obstructive pulmonary disease (COPD) and cystic fibrosis (CF)^1^. Cystic fibrosis is one of the most common recessively inherited rare diseases caused by mutations in the cystic fibrosis transmembrane conductance regulator (CFTR) gene, affecting approximately 160,000 individuals worldwide^2,3^. This progressive and life-limiting disease is characterised by impaired ion transport across the epithelial surface, which leads to an accumulation of thick and viscous mucus in the airways. The altered respiratory environment severely compromises mucociliary clearance, creating a favourable niche for microbial colonization and persistent infections^4^. Chronic inflammation and recurrent infections progressively damage the airway tissue and ultimately lead to respiratory failure - the primary cause of death in around 85% of CF patients^5^.

While the impact of bacterial pathogens, particularly *Pseudomonas aeruginosa* and *Burkholderia cepacia*, on CF lung deterioration and patient morbidity is well established, the role of fungal species in the pathophysiology of CF has been underappreciated and overlooked for decades^6^. Apart from lung infections with *Aspergillus* sp., fungal colonization has traditionally been considered a secondary or incidental event rather than a determinant of disease progression. However, recent large-scale multicenter studies employing metagenomics and advanced molecular techniques have revolutionized our understanding of the CF airway mycobiome^7,8^. These studies have revealed an unexpectedly high fungal diversity and the presence of fungi in relatively high abundance in the CF airways, underscoring the urgent need for in-depth research into fungal pathogenicity in the CF environment and their potential contribution to disease progression^9^.

Individuals with CF are highly susceptible to colonization of the upper (oral cavity) and lower (lung) airways with *Candida* species. Several disease-related risk factors significantly contribute to this susceptibility, including sticky mucus, reduced salivation, elevated glucose (diabetes), prolonged use of antibiotics and inhaled corticosteroids, and, above all, impaired innate immunity^6^. *Candida* species are documented as the most common fungal colonizers of the CF airways, with *C. albicans* representing the most prevalent species identified in expectorated sputum samples^10^. The fungus is present in both paediatric and adult CF patients, with detection rates ranging from 36% to 93%. Chronic lung infection with *C. albicans* can persist for several years, with the fungal load in sputum samples reaching 10³ to 10⁸ cells/mL. Despite its high prevalence, *C. albicans* was dismissed in the past as merely a transient colonizer with minimal inherent virulence, largely attributed to its commensal lifestyle. Over the past decade, however, a growing body of clinical evidence has revealed a strong association between persistent lung colonization with *C. albicans* and disease progression in CF patients^11–13^.

Several clinical studies have reported an association between airway colonization with *C. albicans* and worsening clinical outcomes in CF patients, including declining lung function, prolonged hospitalization and increased morbidity. Notably, it was shown that CF patients have significantly worsened forced expiratory volume in 1 s (FEV1), an index of airway obstruction, after lung infection with *C. albicans*^11,12^. Furthermore, the lung co-colonization with *C. albicans* in the presence of chronic bacterial infection with *P. aeruginosa* or *Staphylococcus aureus* has been associated with rapid deterioration of lung function, more frequent exacerbations requiring hospitalization, and overall worsening of health status in CF patients. In addition to respiratory infections, CF patients are also susceptible to *Candida*-related diseases such as oral thrush and persistent vulvovaginal candidiasis, while systemic candidiasis occurs particularly after lung transplantation or as septicaemia associated with implantable vascular devices^6^. Despite the accumulating evidence, the clinical significance of *C. albicans* in cystic fibrosis remains controversial and a topic of ongoing debate.

Although *C. albicans* is a commensal microorganism in healthy individuals, it can become an opportunistic pathogen in hosts with compromised or dysregulated immune response. This fungus possesses an arsenal of virulence factors that contribute to its ability to infect and persist across different host niches. The key virulence feature is its reversible yeast-to-hypha morphological transition, which plays a crucial role in host tissue invasion, immune evasion, and biofilm formation^14,15^. Biofilms, highly organized communities of microbial cells embedded in an extracellular polymeric matrix, exhibit profound resistance to antifungal therapies, posing a major clinical challenge and often leading to persistent, difficult-to-eradicate infections. Hyphae also produce candidalysin, a cytolytic peptide toxin encoded by the *ECE1* gene. This toxin is a major factor in epithelial damage and is only expressed under hypha-inducing conditions^16,17^.

The combination of these virulence traits—morphological plasticity, biofilm formation and candidalysin production—makes *C. albicans* a pathogen of concern in CF. Moreover, recent studies have identified hyperfilamentous strains of *C. albicans* in CF lungs that harbour disease-specific mutations in regulators of filamentation, allowing them to better adapt to the hostile CF environment and co-exist with opportunistic bacterial pathogens such as *P. aeruginosa*^7,18^.

Despite the increasing awareness of fungal colonization in cystic fibrosis, the virulence traits and pathogenic potential of *C. albicans* within the CF respiratory tract remain unexplored. In particular, the dominant morphological state of *C. albicans* in the CF lung environment is still unresolved, leaving it unclear whether the fungus exists primarily as yeast cells, pseudohyphae, true hyphae, or as part of structured biofilm aggregates. How oxygen fluctuations, associated with CF disease progress and declining lung function, influence the virulence of *C. albicans* is also a question of clinical relevance. This gap in knowledge continues to hamper our understanding of the fungus’ pathogenic role, especially under the complex nutritional and inflammatory landscape of the CF airway. Remarkably, a recent study by O’Meara and colleagues demonstrated that commensal *C. albicans* strains isolated from healthy individuals retain the capacity to cause systemic disease, emphasising that colonization does not necessarily equate to benign coexistence^19^. These findings underscore the urgent need to dissect the virulence mechanisms of *C. albicans* in the context of cystic fibrosis, where a combination of host immune dysfunction and distinct environmental cues may unmask invasive fungal behaviours and drive disease progression.

To address this critical knowledge gap, we investigated clinical *C. albicans* isolates obtained from the sputum of cystic fibrosis (CF) patients with chronic fungal colonization. By integrating in vitro and in vivo approaches, we sought to elucidate the pathogenic potential of these isolates and their adaptive responses to the unique biochemical and immunological landscape of the CF airway. Through this multi-layered approach, our study provides novel insights into the contribution of *C. albicans* to CF lung pathology and highlights fungal virulence as a potentially targetable axis for future therapeutic intervention in CF-related fungal disease.

## Results and Discussion

### CF-mimicking environment sustains the growth and filamentation of Candida albicans

The lung of CF patients (CF airway) represents a dynamic and hostile environment, characterised by well-aerated oxygen-rich regions in patients with preserved lung function, and CO₂-enriched and hypoxic niches emerging as the disease progresses and pulmonary exacerbations become more frequent^20^. These microenvironmental gas shifts together with specific nutritional characteristics and pH of the CF environment can profoundly reshape microbial physiology, virulence, and susceptibility to antifungal therapy.

**Fig. 1:**
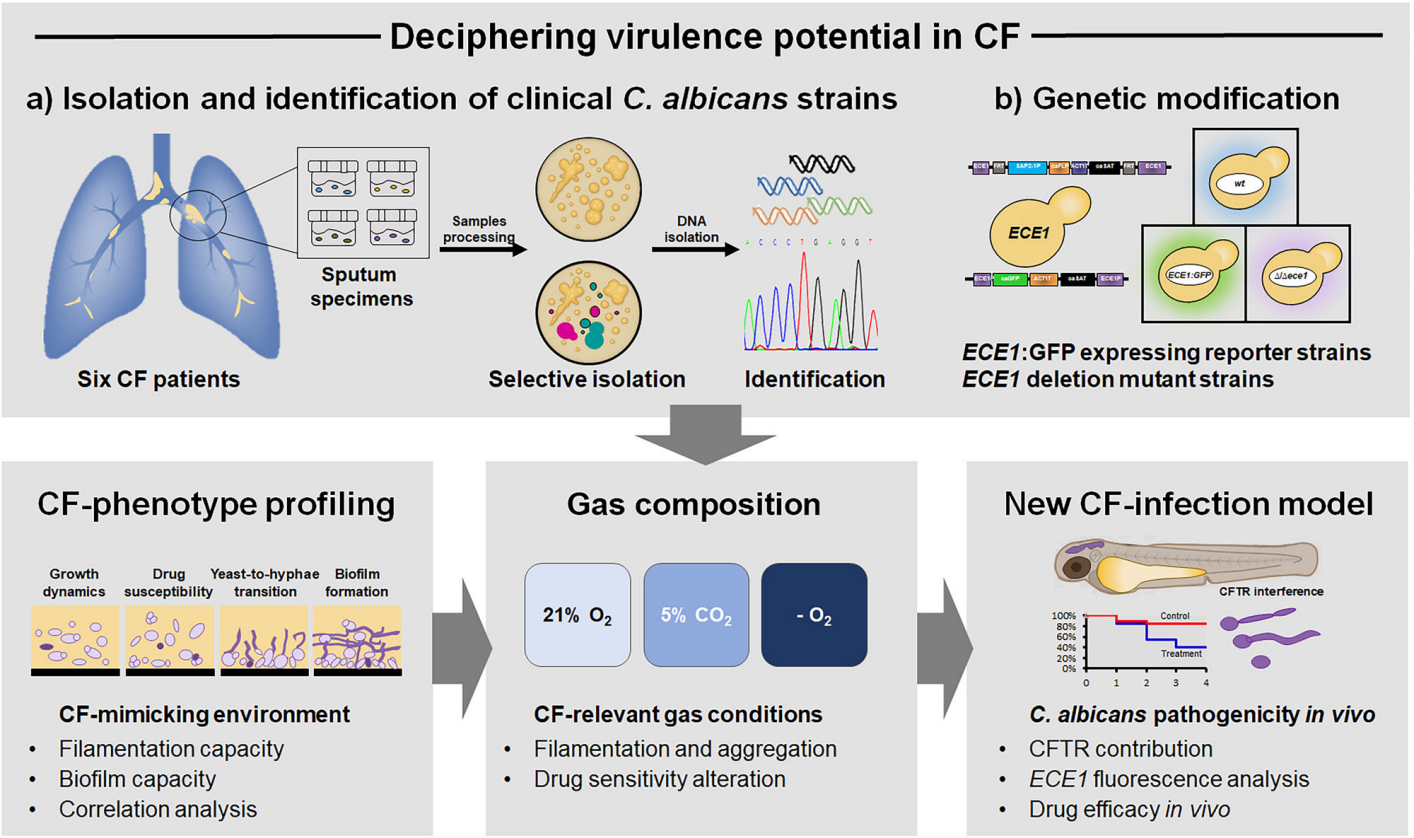
Uncovering the virulence and pathogenic potential of *Candida albicans* colonizing the airways of patients with cystic fibrosis. An overview of the experimental pipeline based on the functionalized CF-related in vitro and in vivo assays. **a** Clinical *C. albicans* isolates were selectively isolated from the sputum of paediatric patients with CF and characterised in vitro. The phenotypic profile of six CF isolates was assessed in SCFM2 and compared with that in several standard media used to simulate the different environmental signals that the fungus may encounter in the human body. **b** Reporter strains expressing *GFP* under the control of the *ECE1* promoter and *ECE1* deletion mutants were constructed and used to evaluate virulence traits under three CF-relevant atmospheric conditions in vitro. A new *C. albicans* infection model was established using zebrafish embryos with *cftr* knockdown (CFTR morphants) to assess the virulence potential of clinical CF isolates, determine their in vivo expression of the candidalysin-encoding *ECE1* gene, and elucidate the role of CFTR in the host’ defence against *C. albicans* infections.

Despite the increasing recognition of the clinical relevance of *C. albicans* in the context of CF, many fundamental aspects of the biology of *C. albicans* in the CF lung environment remain unknown, including the growth capacity, morphological adaptation, pathogenic potential and susceptibility to clinical drugs. To address this gap, we implemented a systematic, multiparametric phenotypic approach, integrating functionalized in vitro and in vivo models to dissect *C. albicans* responses to the key environmental pressures encountered within the CF respiratory tract (Fig. 1).

We first isolated and identified six *C. albicans* strains from the sputum of paediatric CF patients and, for the first time, evaluated their growth under CF-mimicking conditions using Synthetic Cystic Fibrosis medium (SCFM2) (Fig. 1a). SCFM2 is an artificial, nutrient-rich medium composed of mucin, amino acids, extracellular DNA, fatty acids, and carbohydrates, specifically formulated to closely replicate the complex biochemical landscape of CF sputum. This medium is a well-established in vitro model for investigating CF-associated infections, as it mimics the viscoelastic properties of expectorated CF sputum and supports microbial transcriptional and growth profiles that closely resemble those observed in vivo^21,22^. However, as SCFM2 contains both filamentation-inducing components (amino acids, GlcNAc, and glucose)^23,24^ and filamentation-inhibitory macromolecules (notably mucin)^25^, alongside molecules with unclear roles in morphogenesis (e.g., DOPC, DNA), we sought to determine whether *C. albicans* retains its ability to undergo hyphal development in this complex artificial medium. Filamentation in SCFM2 was compared to filamentation in several other media (RPMI, SLAD, Spider, Lee, and GlcNAc) mimicking diverse environmental cues that *C. albicans* may face when colonizing the different niches of the human body^26^.

**Fig. 2:**
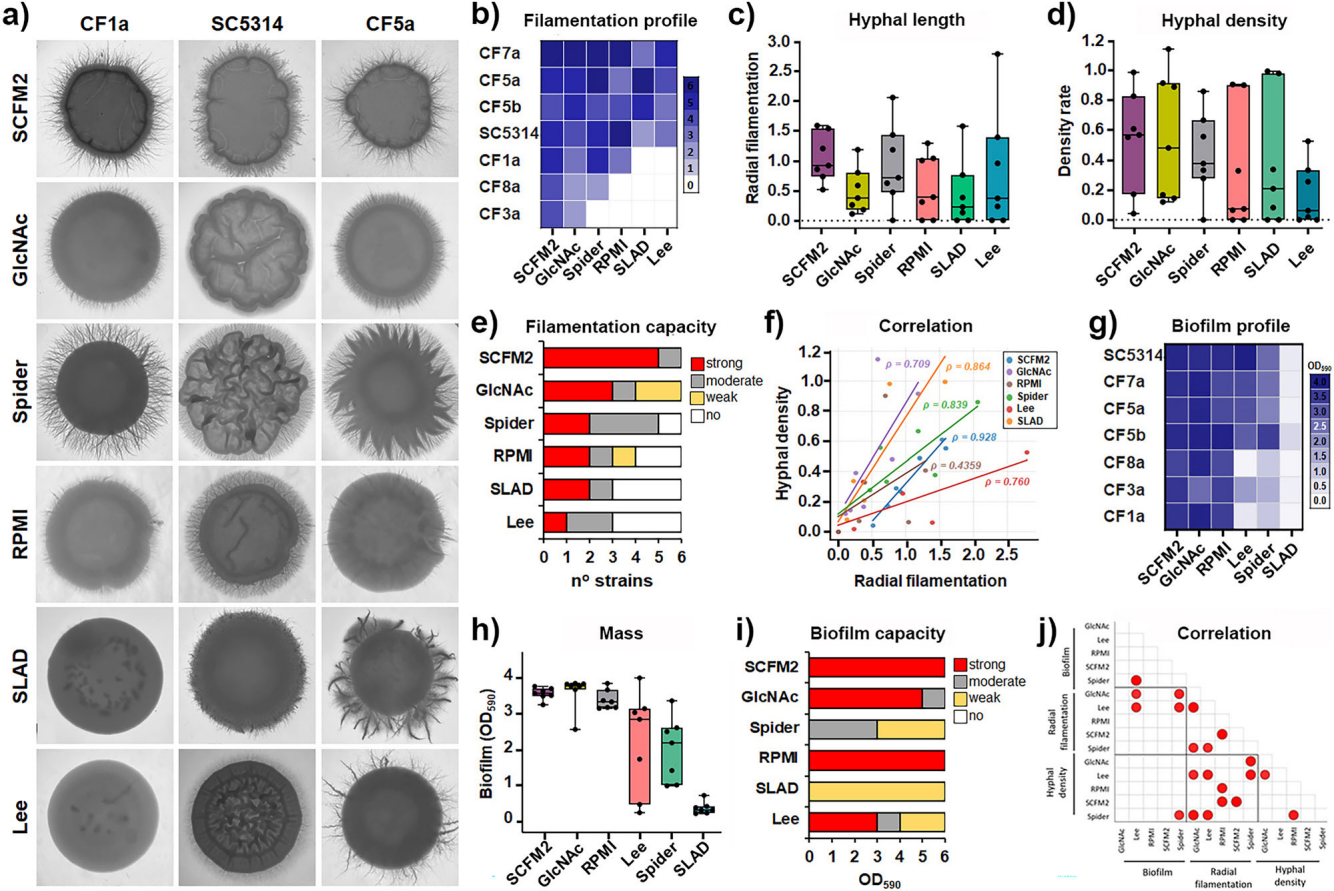
Clinical isolates of *Candida albicans* retain the capacity to undergo filamentation and biofilm formation across a range of environmental conditions. **a** Clinical CF isolates were examined for their filamentation ability in different media, including SCFM2. Representative colony images from three independent experiments are shown. The reference strain SC5314 was used as control. **b** Colour-based heatmap showing the different filamentation profile among CF isolates across tested media, assessed by **c** radial filamentation and **d** hyphal density, quantified to capture filamentation-related phenotypes. Each spot indicates an individual CF isolate and represents the mean value of three independent measurements. **e** Filamentation capacity of clinical CF isolates in different media, categorized by radial filamentation and hyphal density, showing that SCFM2 is the strongest inducer of filamentation. **f** Correlation analysis between filament length and hyphal density, performed for each medium using Spearman’s rank correlation, revealing the strongest positive association in SCFM2. Linear trend lines are shown for visualization only. **g** Colour-coded heatmap demonstrating the ability of all clinical CF isolates to form biofilms under various environmental cues **h** determined by measuring biofilm mass (CV assay) (n = 6). **i** Biofilm-forming capacity of CF isolates across different media categorized by the following criteria: OD_590_ > 3 (strong), 1 < OD_590_ ≤ 3 (moderate), 0.2 < OD_590_ ≤ 1 (low), OD_590_ ≤ 0.2 (non-biofilm forming). **j** Correlation analysis between biofilm-related characteristics **–** biofilm mass, radial filamentation and hyphal density, represented by Pearson’s correlation. Red spot indicates statistically significant correlation p < 0.05.

To assess the filamentous growth of CF isolates, we employed the standard agar plate-based assay that simultaneously enables measurement of colony diameter, radial filamentation (average filament length) and hyphal density (Fig. 2a–e). Radial filamentation was quantified by calculating the relative expansion of hyphal outgrowth from the colony centre, as recently described^27^, providing a high-resolution, pixel-based measure that captures the full extent of filamentous projections. We observed striking inter-strain and inter-medium variability in filamentation phenotypes among clinical CF isolates, ranging from robust filamentation in SCFM2 to a complete absence of radial filaments in some other media (Fig. 2a–c). Notably, even the isolates that failed to form hyphae in RPMI, Spider, SLAD or Lee media developed vigorous hyphae in SCFM2 (Fig. 2a, b), underscoring the strong filamentation-inducing potential of CF-mimicking conditions. Moreover, comparative analysis across all conditions (media) tested showed that SCFM2 supported the development of longest filaments (P < 0.01, Fig. 2c), while the highest hyphal density was induced in GlcNAc medium (P < 0.01, Fig. 2d). Importantly, both media contain *N*-acetyl glucosamine (GlcNAc), which is a highly abundant component of CF sputum^28^. Based on these two filamentation-related features, we found that the highest filamentation capacity was in SCFM2 (Fig. 2e). Given the heterogeneity in filamentation phenotypes observed across different media, we employed non-parametric Spearman’s rank correlation to more robustly assess the association between radial filamentation and hyphal density. Correlation analysis performed for each medium revealed the strongest positive association in SCFM2 (ρ = 0.93, *p* = 0.0025; Fig. 2f), compared to other media.

Considering the well-established relationship between filamentous growth and biofilm development in *C. albicans*^29^, we next systematically assessed the biofilm-forming capacity of clinical CF isolates under multiple environmental conditions (different media) (Fig. 2g), and compared with that of the reference strain *C. albicans* SC5314, known for its robust biofilm-forming phenotype^30^. We found that all clinical isolates, regardless of their filamentation capacity in vitro, formed biofilms to some extent under each of the six tested conditions (Fig. 2g, h), demonstrating the adaptability of CF strains to various host-relevant stimuli. The extent of biofilm formation, however, varied considerably between media, reflecting the influence of specific nutrients. (Fig. 2h). We found that CF isolates consistently produced the highest biofilm biomass in SCFM2 and GlcNAc (Fig. 2h, i), media enriched in GlcNAc. Further quantitative analysis showed that CF isolates formed 3.3-fold, 14.9-fold and 18.7-fold higher biofilm mass in SCFM2 medium than in Spider, Lee or SLAD media (Figure S1), underlining the role of nutrient composition in regulating biofilm development. In support of this, replacing glucose with GlcNAc (Lee medium vs. GlcNAc medium) led to up to 17.3-fold increase of biofilm mass in some isolates, underscoring the biofilm-inducing effect of GlcNAc. GlcNAc, a degradation product of mucins and bacterial cell wall components, is highly abundant in the inflamed CF airway and certain niches in the digestive tract. It serves not only as a nutrient but also as a signalling molecule that promotes morphogenesis, biofilm formation, and virulence in *C. albicans*^31^.

We also observed a positive correlation between filamentation and biofilm formation, with CF strains exhibiting strong filamentation capacity also forming robust biofilms (Fig. 2j), which is in line with previous studies^32^. Notably, the isolates that formed strong biofilms (OD ≥ 3) are characterised with radial filamentation values > 0.5 and/or hyphal density values > 0.3 in the same media conditions (Figure S2). Biofilm production in SCFM2 by clinical CF isolates was comparable to that of the reference strain *C. albicans* SC5314, a well-established biofilm-forming bloodstream isolate. However, it is important to emphasise that even isolates CF8a and CF3a, characterised by low filamentation capacity and the absence of hyphae in Lee, SLAD, RPMI and Spider media, successfully formed biofilms in each of these media, highlighting that biofilm formation is not strictly dependent on extensive hyphal growth.

Collectively, these findings demonstrate that clinical *C. albicans* isolates from CF patients are highly responsive to nutrient signals characteristic of the CF lung, with amino acid- and GlcNAc-rich environments, such as SCFM2, strongly promoting robust biofilm formation. This emphasises the remarkable adaptive and virulence potential of these isolates under physiologically relevant, host-mimicking conditions. To our knowledge, this is the first systematic in vitro study to assess the biofilm-forming capacity of *C. albicans* directly isolated from CF sputum using the medium that recapitulates key aspects of CF mucus - SCFM2. Moreover, the consistent ability of CF strains to form biofilms in SCFM2 and GlcNAc media highlights the importance of host-derived nutrients - including amino acids, mucins, and GlcNAc - in supporting fungal persistence. These components, abundant in the inflamed and mucus-filled CF airway, likely act as potent inducers of biofilm formation and chronic colonization. While earlier studies have investigated *C. albicans* biofilm formation in a medium resembling SCFM2, they lacked critical components such as mucin and di-oleoyl phosphatidylcholine (DOPC), both abundant in CF sputum, and were performed using laboratory strains rather than clinically relevant CF isolates^33^. Our study overcomes these limitations by modelling a more physiologically accurate CF airway environment and using CF patient-derived isolates, thereby providing novel insight into nutrient-driven biofilm formation in a clinically relevant context. Data obtained here also suggest that disrupting nutrient sensing, GlcNAc uptake, or biofilm matrix assembly may offer novel therapeutic strategies to reduce fungal burden and alleviate inflammation in CF-associated *C. albicans* infections.

### The gas-nutrient interplay governs the growth and filamentation of C. albicans in CF-like environment

**Fig. 3:**
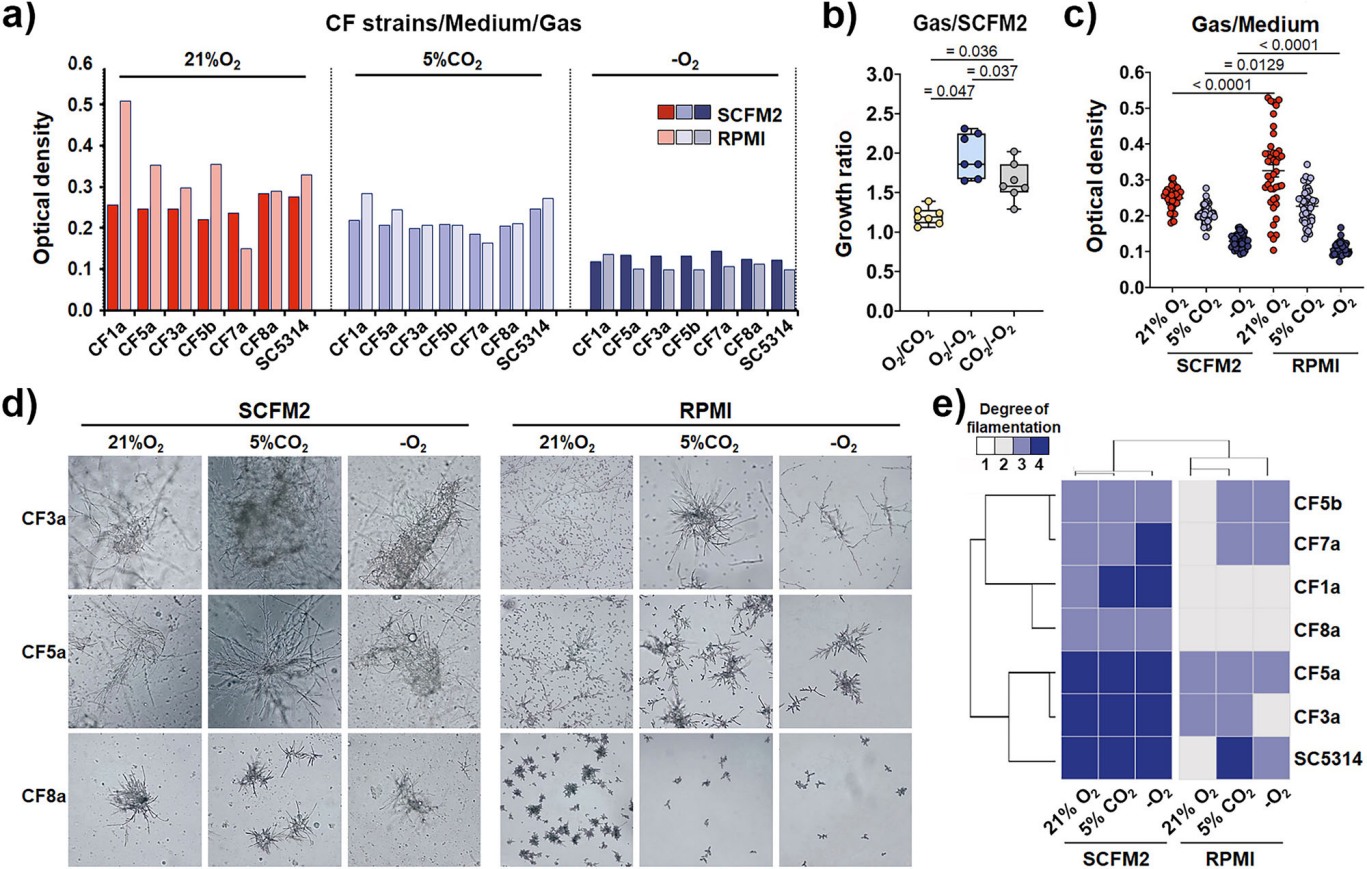
CF-mimicking environment modulates growth and filamentation patterns of clinical isolates of *C. albicans* from CF patients. **a** Growth of CF isolates (n = 6) and the SC5314 reference strain in SCFM2 and RPMI media under the three CF-relevant gas conditions. Data are presented as mean ± standard deviation from three independent experiments performed in duplicate or triplicate for each isolate. **b** Changes in the growth rate of clinical CF isolates in SCFM2 medium depending on the environmental gas composition. Each dot represents the mean growth value of an individual isolate, derived from two independent experiments, performed in triplicate. **c** Growth comparison of the clinical CF isolates under 21% O_2_, 5% CO_2_ and hypoxic conditions in SCFM2 versus RPMI. Each dot corresponds to the growth value of an individual isolate (six values per isolate). **d** Filamentation phenotype (length of filaments and aggregation pattern) of CF isolates influenced by both gas and nutritional conditions. **e** Colour-coded heatmap presenting hierarchical clustering of *C. albicans* isolates based on their filamentation phenotypes under various gas and nutritive conditions. Clustering was performed using Ward’s linkage method with Euclidean distance, showing distinct filamentation profiles in SCFM2 versus RPMI medium.

Given that dynamic fluctuations in oxygen availability characterise CF airways throughout disease progress^34^, we sought to determine how different gas conditions affect the growth of *C. albicans* isolates in CF-mimicking environment. We cultured CF isolates in SCFM2 under ambient air (21% O₂), enriched CO₂ (5%), and hypoxic (low O₂) conditions, and found that all isolates were capable of growing under all three gas conditions (Fig. 3a, b, Figure S3). However, comprehensive statistical analysis revealed that gas composition significantly influenced the growth of clinical CF isolates (Friedman test, p = 0.00091; Fig. 3b), with higher proliferation in ambient air compared to 5% CO₂ (p = 0.047), and hypoxia (p = 0.036) (Fig. 3b). These findings show that both oxygen and CO_2_ availability significantly influence *C. albicans* proliferation in CF-mimicking environment, suggesting they could act as key modulators of fungal growth in the CF lung. Moreover, this gas-dependent growth trend was also conserved in the reference strain SC5314 and was independently confirmed for each individual clinical CF isolate (Friedman tests, p < 0.05).

To determine whether the observed gas-dependent growth pattern was specific to SCFM2 or reflects a broader physiological phenomenon, we conducted parallel experiments in RPMI medium and also observed a similar trend (O₂ > CO₂ > hypoxia; p = 0.0021, Friedman test) (Fig. 3c), confirming that the gas-responsive growth behaviour is not restricted to SCFM2. Notably, the extent of gas-driven growth differences was more pronounced in RPMI, suggesting that the interplay between gas composition and nutritional environment further modulates fungal proliferation. While growth under 21% O_2_ and 5% CO_2_ conditions was significantly higher in RPMI than in SCFM2 (P < 0.05), clinical CF isolates exhibited greater growth in SCFM2 under hypoxic conditions (P < 0.0001, Fig. 3c). These data demonstrate that oxygen and carbon dioxide availability or limitation significantly influence the growth capacity of *C. albicans* isolates from CF patients. In light of the well-established spatial and temporal heterogeneity in airway oxygenation across CF lung compartments and between patients, in particular with the disease progression^34^, our findings suggest that the dynamic fluctuations in gas composition could be a critical microenvironmental factor in shaping fungal colonization patterns, proliferation potential, and overall fungal burden in the CF respiratory tract. Considering that *C. albicans* possesses gas-responsive physiological adaptations that influence its growth, morphology, and aggregation, we next assessed whether oxygen and carbon dioxide fluctuations in a CF-like environment directly modulate the yeast-to-hypha switch - a hallmark of *C. albicans* pathogenicity. Since filamentation is tightly linked to virulence, immune evasion, tissue invasion and inflammation, uncovering how CF-relevant gas conditions regulate fungal morphogenesis is critical for understanding its pathogenic potential in the CF lung and potential role in disease progression. This prompted us to investigate further whether the gas fluctuations, characteristic of the CF airway, serve as regulatory cues that modulate the morphogenetic program of *C. albicans* and, consequently, influence its pathogenic potential.

Using a semi-quantitative scoring approach (Figure S4), we analysed the filamentation patterns of clinical isolates from CF patients under three distinct gas conditions - ambient air (21% O₂), enriched CO₂ (5%), and hypoxia, and compared their morphogenesis responses in SCFM2 and RPMI (Fig. 3d, e). Remarkably, we found that clinical CF strains retained the ability to form filaments not only under ambient air, but also under hypoxic conditions, underscoring their preserved intrinsic filamentation potential. These findings indicate that the morphogenetic competence of CF isolates remains intact despite fluctuations in O_2_ or CO₂ levels. Importantly, SCFM2 consistently supported more robust filamentation across all gas environments compared to RPMI, reinforcing its capacity to intrinsically mimic the pro-filamentation signals found in CF sputum and thereby promote filamentous growth in *C. albicans*.

However, we also observed that the filamentation phenotype among CF isolates - specifically filament length and clustering pattern - was strongly influenced by the gas environment (Fig. 3d, Figure S4). Hypoxia, followed by 5% CO₂, consistently induced more robust filamentous growth and aggregative behaviour than the ambient 21% O_2_ condition (Fig. 3e). In SCFM2, all tested *C. albicans* strains produced long, intertwined filaments that often coalesced into tuft-like or aggregate-like clusters, particularly under hypoxic conditions (Fig. 3d, Figure S4). In contrast, RPMI favoured the development of shorter, yet more highly branched structures (Fig. 3d, Figure S4). These findings suggest that hypoxic CF microenvironments, and to a lesser extent CO₂-enriched niches^35^, both characteristic of CF lung mucus plaque, may act as the morphogenetic cues that promote filamentation in *C. albicans* in nutrient-rich mucus-like conditions, as modelled by SCFM2. Furthermore, while nutrient composition clearly influenced the overall filamentation potential (SCFM2 vs. RPMI, Fig. 3e), hyphal length and colony morphology exhibited substantial strain-specific variability. Individual clinical isolates displayed distinct filamentation phenotypes even under identical environmental conditions, highlighting the influence of genetic background in modulating morphogenetic outcomes.

Collectively, these results underscore the critical interplay between environmental gas availability and nutrient context in shaping fungal morphogenesis in CF-relevant settings. They further emphasise the necessity of using disease-relevant media such as SCFM2 and controlled gas conditions to accurately assess the virulence characteristics of CF clinical isolates. Such approaches are essential for predicting the pathogenic behaviour and therapeutic response of *C. albicans* in the chronic infection environment characteristic of cystic fibrosis.

### CF-mimicking environment decreases the drug susceptibility of clinical isolates of C. albicans

Based on these findings, we next hypothesized that the unique chemical composition and gas microenvironment of CF sputum may reduce *C. albicans* susceptibility to antifungal agents and thereby limit the translational value of current in vitro testing systems. Specifically, to investigate whether the CF lung environment compromises the efficacy of antifungals and how gas shifts influence the sensitivity of fungi to clinical drugs, we questioned the clinical relevance of standard antifungal susceptibility testing protocols recommended by CLSI or EUCAST guidelines, which are typically performed in RPMI medium under normoxic conditions^36^.

We first evaluated the antifungal activity of nystatin under standard aerobic conditions by comparing its minimum inhibitory concentration (MIC) in SCFM2 and RPMI media and found that MIC values in RPMI medium ranged from 0.5-1 µM, indicating that tested CF isolates are susceptible to nystatin (data not shown). However, MIC values in SCFM2 were up to 4-fold higher than in RPMI (1–4 µM vs. 0.5–1 µM), indicating that CF-like nutritional conditions significantly reduce the efficacy of nystatin. This reduction was further exacerbated by altered gas composition. Specifically, under hypoxic conditions, the MIC of this drug for some isolates exceeded 4 µM in SCFM2, suggesting that oxygen limitation, which is common in the mucus-clogged CF airways, further reduces the susceptibility of clinical isolates of *C. albicans*. Similar hypoxia-governed drug susceptibility was also observed in RPMI. These data suggest that CF environment itself diminishes antifungal drug activity, an effect that is further exacerbated under oxygen-limited conditions, ultimately reducing the efficacy of applied therapies. While previous studies have demonstrated decreased antibiotic efficacy against *Mycobacterium abscessus*^37^ and *P. aeruginosa*^38^ in CF-mimicking media, no study to date has evaluated the activity of clinically used antifungal agents against *C. albicans* under such conditions - particularly not in the context of variable gas environments and in SCFM2 medium.

**Fig. 4:**
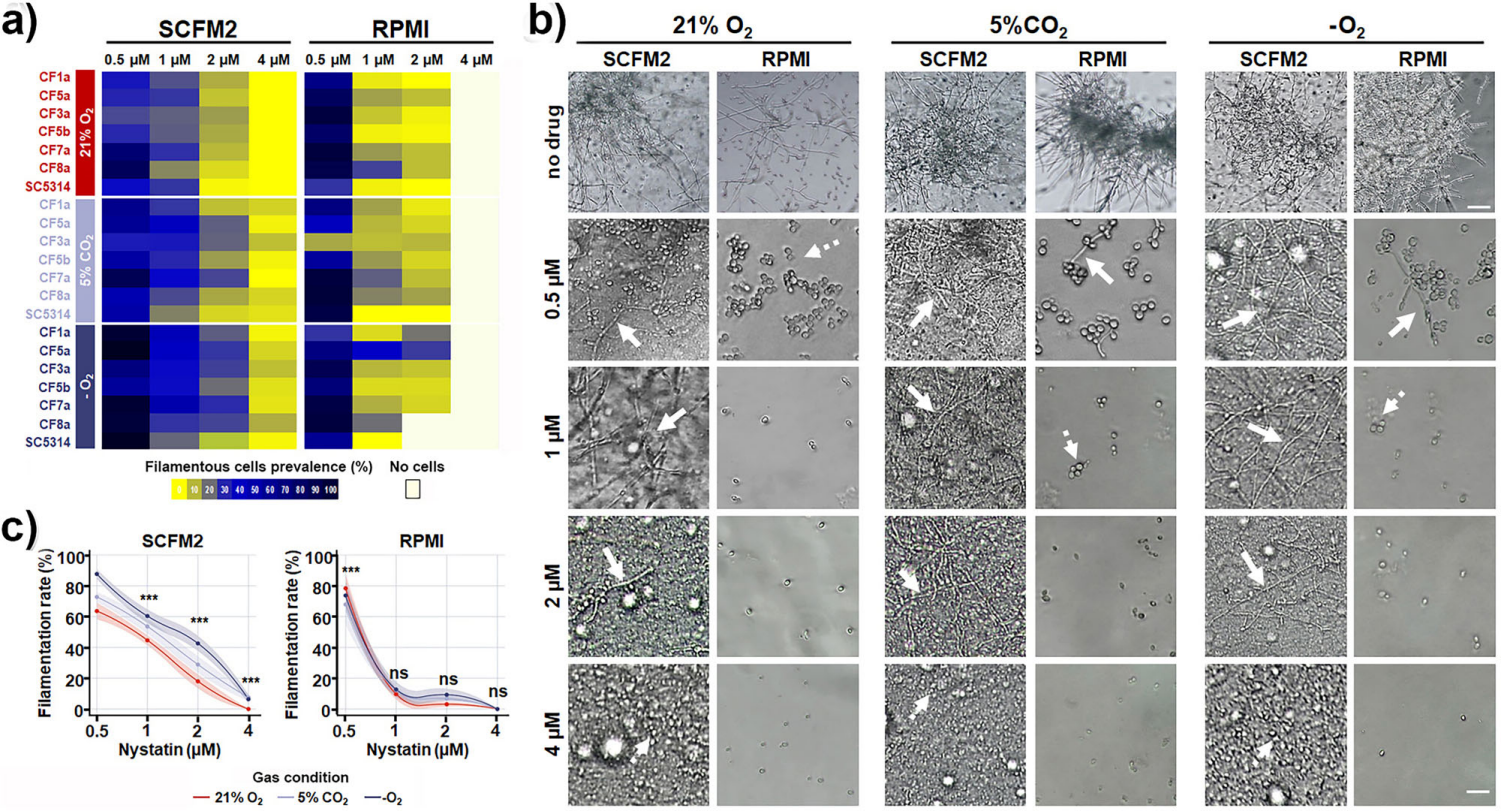
CF-mimicking environment decreases the susceptibility of clinical isolates of *C. albicans* from CF patients to the antifungal drug nystatin. **a** Drug-response heatmap showing the effect of nystatin upon different gas and nutritive conditions on the prevalence of filamentous cells in each clinical CF isolate (average of three independent experiments). **b** Representative images of the dose-dependent effect of nystatin on cell morphology of isolate CF5a in SCFM2 versus RPMI medium. Dashed arrow indicates yeast-like cells, while arrow indicates filaments. **c** Gas composition significantly influences filamentation-inhibitory effect of nystatin (proportion of filaments), as assessed in SCFM2 and RPMI medium. One-way ANOVA with the Bonferroni test was used to determine statistical significance between groups. ***, P < 0.001.

Consistent with these results, we found that the filamentation-inhibitory activity of nystatin was significantly attenuated in SCFM2 and further modulated by the prevailing gas conditions (Fig. 4a). We noticed that nystatin was markedly more effective in suppressing filamentation of clinical CF isolates in RPMI compared to SCFM2 (Figure S5); while the drug effectively inhibited filamentation at 0.5 and 1 µM doses in RPMI, hyphae remained clearly visible even at 2 µM in SCFM2 (Fig. 4b). Moreover, hyphal growth was substantially robust under CO₂ and especially under hypoxic conditions, with the more pronounced effect in SCFM2 (Fig. 4b, c), highlighting the critical influence of nutrient-gas interplay on drug susceptibility. Crucially, when we performed efficacy testing under standard conditions (RPMI, 21% O₂), we observed complete inhibition of filamentation at 0.5 µM nystatin; however, short filaments still emerged in the same medium when either CO₂ or hypoxia was introduced (Fig. 4b). These findings indicate that evaluating antifungal efficacy solely under conventional testing conditions may fail to capture the true *C. albicans* response to clinical treatment, and underscore the importance of assessing drug activity under physiologically relevant conditions, including elevated CO₂ and low oxygen.

Collectively, our findings highlight the critical interplay between environmental gas composition and nutrient context in shaping the morphogenetic behaviour and antifungal susceptibility of *C. albicans* in CF-relevant conditions. We demonstrate that the CF lung-mimicking environment, characterised by altered oxygen and CO₂ levels, markedly diminishes both the antifungal and anti-virulence efficacy of nystatin. These results raise important concerns about the clinical relevance of conventional susceptibility testing, as standard media and normoxic conditions may significantly overestimate therapeutic effectiveness. In light of the poor correlation frequently observed between in vitro susceptibility and clinical outcomes in CF patients, and the need for long-term antifungal therapy, our data underscore the urgent need for physiologically and disease-relevant testing platforms. The use of models that incorporate CF-specific nutritional and gas conditions, such as SCFM2 and O₂/CO₂/hypoxia, will be essential for accurately assessing the pathogenic potential and optimizing antifungal treatment strategies in CF-associated fungal infections.

### CFTR deficiency enhances CF host susceptibility to Candida albicans infection and candidalysin-driven pathogenesis

**Fig. 5:**
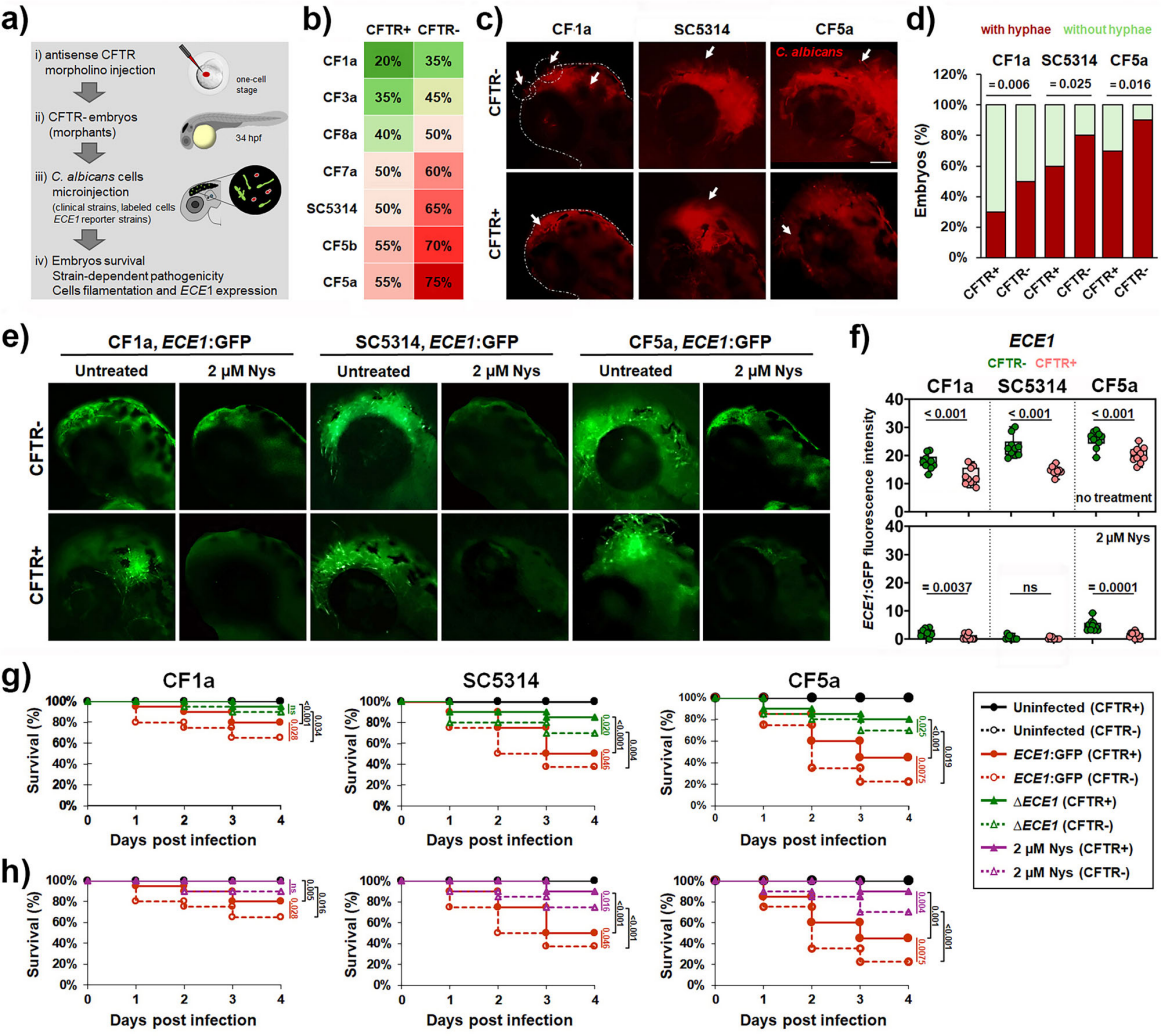
CFTR-deficient zebrafish embryos reveal enhanced *Candida albicans* virulence, increased *ECE1* expression, and decreased therapeutic efficacy of nystatin in vivo. **a** Schematic overview of the newly developed *C. albicans* infection model using CFTR-deficient (CFTR^-^) embryos, generated via CFTR knockdown, to mimic the CF host environment. **b** Colour-coded map showing the survival rates of CFTR^+^ and CFTR^-^ embryos (n = 20) at 120 h post-fertilisation (hpf), following infection with *C. albicans* isolates derived from the sputum of CF patients. **c** Representative fluorescence images of infected embryos showing extensive hyphal growth (arrow) within tissues and epithelial barrier breaching in the head region (dashed outline). The SC5314 reference strain was used as control. Fungal cells were labelled with a red cell tracker. **d** Proportion of CFTR^-^ versus CFTR^+^ embryos with or without visible hyphae in the hindbrain at 24 h post-infection (hpi), assessed by fluorescence microscopy (n = 20). Statistical significance was evaluated using the χ2 test by comparing the distribution of embryos with and without hyphae between CFTR^+^ and CFTR^-^ groups. **e** in vivo fluorescence of *ECE1:*GFP reporter strains upon infection of CFTR^-^ and CFTR⁺ zebrafish embryos, with or without nystatin treatment. **f** Quantification of GFP fluorescence intensity as a measure of *ECE1* expression under the same conditions. **g** Contribution of *ECE1* to *C. albicans* pathogenicity assessed by Kaplan–Meier survival analysis of CFTR^-^ and CFTR⁺ embryos infected with either *ECE1*:GFP reporter strains or *ECE1* deletion mutants. **h** Validation of the newly established infection model by assessing the antifungal efficacy of nystatin at 2 µM. Survival differences between CFTR⁺ and CFTR^-^ embryos under each condition were statistically analysed using the log-rank (Mantel–Cox) test. Data for the *ECE1:GFP* strain are identical in panels g and h, as both analyses were performed within the same experiment. In panel g, *ECE1:GFP* serves as a reference strain for the deletion mutants, while in panel h it represents the untreated control for the nystatin-treated groups.

Despite growing evidence that *C. albicans* clinical isolates derived from cystic fibrosis patients possess the ability to form filaments under CF-mimicking conditions in vitro, it remained unclear whether these strains possess true pathogenic potential in vivo and whether a CF-specific host environment influences fungal virulence. To address this gap, we developed a novel in vivo model of *C. albicans* infection using CFTR expression-deficient zebrafish (*Danio rerio*) embryos (Fig. 5a), thereby mimicking the CF host milieu.

We leveraged the optical transparency and genetic tractability of the zebrafish embryo and employed a morpholino-based knockdown strategy to generate CFTR-deficient (CFTR⁻) morphants. This approach simulates CF-like conditions and has previously been validated for modelling CF-associated infections with *P. aeruginosa* and *Mycobacterium spp*.^39,40^. To investigate whether CFTR depletion increases host susceptibility to *C. albicans* infection and whether CF clinical isolates exhibit enhanced expression of the virulence traits in the CFTR-deficient host, we injected CFTR⁺ and CFTR⁻ embryos with 55–65 yeast cells of each clinical isolate - a dose previously established to induce ~50% mortality of wild-type embryos by the reference SC5314 strain, which was used here as the positive control^41^.

We found that CFTR⁻ morphants exhibited higher mortality compared to their CFTR⁺ counterparts across all tested isolates (Fig. 5b, Figure S7). These results clearly indicate that CFTR plays a protective role against invasive fungal infection and that CFTR deficiency increases host susceptibility to *C. albicans* infections. Furthermore, microscopic analysis revealed an increased prevalence of filamentation in CFTR⁻ embryos, with more pronounced hyphal invasion and deeper epithelial tissue penetration (Fig. 5c, d). Embryos infected with highly filamentous isolates (e.g., CF5a and CF7a) displayed the highest mortality rates, while those infected with less filamentous isolates (e.g., CF1a and CF8) survived longer and showed restricted fungal spread. Remarkably, we observed that these strains failed to breach epithelial barriers despite forming dense filamentous networks at the infection site (Fig. 5c), suggesting that epithelial invasion may require not only filamentation but also the production of candidalysin or additional invasive factors. Correlation analysis further revealed the strong association between filamentation capacity of isolates in SCFM2 medium (mimicking CF sputum) and their ability to induce mortality in CFTR⁻ embryos (P < 0.01 for all tested isolates). These findings underscore the utility of this model for evaluating strain-specific pathogenicity and suggest that the CF environment exacerbates virulence traits in *C. albicans*.

Given that filamentation was enhanced in CFTR-deficient embryos, we next asked whether the fungal toxin candidalysin is expressed during infection and contributes to the increased mortality of CFTR morphants. Candidalysin, a pore-forming peptide encoded by the *ECE1* gene, is secreted during hyphal growth and plays a critical role in epithelial damage and immune evasion across multiple host systems, including zebrafish embryos^16^. To assess its expression in the CF host environment, we generated GFP-reporter strains of two clinical isolates (less filamentous CF1a and more filamentous CF5a) and the SC5314 reference strain, placing GFP under the control of the *ECE1* promoter. Parental clinical strains were selected based on their virulence properties, with one more and one less virulent strain compared to SC5314 according to both in vitro and in vivo assays. Following infection of CFTR⁻ embryos, we observed strong GFP fluorescence at the infection sites (Fig. 5e, f, Figure S7), indicating active in vivo expression of *ECE1* in the CF host. These reporter strains retained virulence comparable to their parental strains, confirming that insertion of the reporter cassette did not compromise infectivity. Notably, using these reporter strains, we observed increased fluorescence intensity, indicative of higher *ECE1* expression, in CFTR- compared to CFTR+ embryos. These findings indicate both enhanced *C. albicans* pathogenicity and increased candidalysin production in CF-like environment.

To further confirm that embryo lethality correlates directly with the pathogenic potential of *C. albicans* isolates, we infected the embryos with the *ECE1* deletion mutants carrying homozygous deletions of both *ECE1* alleles. Consistent with our hypothesis, embryos infected with these mutants displayed significantly lower mortality compared to those infected with the corresponding wild-type or reporter strains (Fig. 5g). Although mortality was reduced in the absence of *ECE1*, it remained consistently higher in CFTR⁻ embryos than in CFTR⁺ counterparts, further indicating that both fungal virulence and host CFTR status contribute to disease severity. These findings clearly demonstrate that candidalysin enhances the lethality of *C. albicans* and directly implicate candidalysin in the pathogenesis of *C. albicans* infection in the CF host.

To evaluate the potential of this new in vivo platform for antifungal drug screening, we exposed infected embryos to nystatin, a polyene antifungal with proven efficacy in zebrafish-*Candida* infections^41,42^. Both CFTR⁻ and CFTR⁺ embryos treated with nystatin exhibited markedly improved survival (Fig. 5h) and reduced fungal burdens (P < 0.01), as quantified via GFP fluorescence (Fig. 5f). Remarkably, nystatin treatment appeared less effective in CFTR morphants compared to CFTR+ embryos, suggesting not only increased susceptibility of CFTR- embryos to *C. albicans*, but also reduced antifungal efficacy of nystatin in the CF host environment. These findings indicate that CFTR deficiency may impair host responsiveness to antifungal therapy, highlighting the need to consider host context in treatment strategies for *C. albicans* infections in CF patients.

To our knowledge, this is the first study to develop and functionally validate a zebrafish model of fungal infection within the pathophysiological context of cystic fibrosis. Previous zebrafish CF models have focused on infections with pathogenic bacteria such as *P. aeruginosa*^39^*, M. abscessus*^43^, and *M. fortuitum*^40^. By integrating multiple key parameters such as CFTR deficiency, fungal morphological plasticity, *ECE1*/candidalysin expression, and therapeutic responsiveness - into a single tractable in vivo system, we have established a powerful platform for in-depth investigation of CF-associated fungal disease. Importantly, the model accurately recapitulates key aspects of fungal disease progression in CF, bridging the gap between in vitro virulence trait profiles and in vivo infection outcomes. This animal model provides new opportunities to investigate CF host-*Candida* interactions, particularly how CFTR dysfunction modulates innate immune responses, including fungal recognition, chemotaxis, and phagocytic clearance by CFTR-deficient neutrophils and macrophages. Such insights are critical for understanding the immunopathology of fungal infections in CF and for the development of targeted antifungal and immunomodulatory therapies.

Beyond its mechanistic utility, the newly established zebrafish CF-*Candida* infection model holds strong translational potential. It enables *high-throughput* in vivo drug screening under CF-relevant conditions, supporting the preclinical discovery and prioritization of novel, effective, and safe antifungal and anti-virulence agents, including those targeting candidalysin production or activity. This represents a step forward from conventional antifungal strategies by embracing the complexity of host-pathogen interactions in CF.

Taken together, our study provides compelling experimental evidence that *C. albicans* strains isolated from CF sputum are not merely benign, transient colonizers of the CF airway, but rather present environmentally responsive and virulent pathogens. We reveal that the behaviour of these strains is profoundly shaped by the distinctive nutritional and gas conditions of the CF lung. By combining functional in vitro and in vivo models that recapitulate the key features of the CF environment, we demonstrate that clinical isolates from CF patients exhibit remarkable virulence potential and adaptability, characterised by robust filamentation, persistent biofilm formation, and sustained growth under low oxygen and elevated CO₂ levels, characteristic of the diseased CF airway. Furthermore, results from a newly established zebrafish CF infection model indicate that *C. albicans* virulence is exacerbated in CFTR-deficient hosts and accompanied by elevated *ECE1* expression, likely reflecting increased candidalysin production. Notably, our findings also reveal that CFTR deficiency not only heightens host susceptibility to fungal infection, but also compromises the efficacy of antifungal treatment in vivo, underscoring the critical role of host physiology in determining infection outcome.

While a growing body of clinical evidence supports the role of *C. albicans* in disease progression and the decline of respiratory function in patients with CF^11–13^, its pathobiology, the spectrum of expressed virulence traits, and its susceptibility to clinical therapy within the unique microenvironment of the CF lung have remained entirely unexplored to date. In current clinical practice, the detection of *C. albicans* in CF sputum is generally not considered sufficient to warrant antifungal treatment, as its presence is typically interpreted as airway colonization rather than active infection, except in cases of clinical deterioration, such as decline in lung function, or in patients with advanced stages of the disease. Current available CF management guidelines, including those from the UK’s National Institute for Health and Care Excellence (NICE, NG78) and the USA Cystic Fibrosis Foundation, outline therapeutic recommendations primarily targeting *Aspergillus fumigatus*, but notably lack guidance for managing *Candida* infections^44,45^. Similarly, the Clinical Guidelines of the Royal Brompton Hospital (RHB) on care of children with cystic fibrosis^46^ describe *Candida* as a frequent finding in sputum, likely due to oral contamination (often driven by long-term antibiotic use), and recommend antifungal therapy (i.e. nystatin or miconazole) only in cases of oral candidiasis. Oral fluconazole or intravenous antifungal agents are considered if *Candida* is detected in broncho-alveolar lavage fluid.

Utilizing SCFM2, a chemically defined medium that closely mimics the nutritional composition of CF sputum, we further demonstrate that specific nutrients, particularly GlcNAc and amino acids, synergize with oxygen depletion to enhance hyphal growth and aggregate formation, while concurrently diminishing antifungal drug efficacy. This interplay between nutrient availability and gas composition was found to critically modulate fungal behaviour, especially under nystatin exposure, where standard susceptibility testing conditions markedly overestimated antifungal activity. These findings challenge the clinical relevance of conventional antifungal susceptibility testing and underscore the urgent need to re-evaluate current protocols by incorporating CF-relevant media and gas conditions.

While nystatin was used in this study as a model fungicidal agent to benchmark antifungal efficacy under CF-relevant conditions, it is important to note that this drug is not routinely employed for the treatment of pulmonary *Candida* infections. However, an intravenous formulation of a nystatin derivative (BSG005), intended for the treatment of invasive fungal infections, is in clinical trials (ClinicalTrials.gov, Identifiers: NCT04921254 and NCT06678113). While BSG005 exhibits broad-spectrum antifungal activity, even against azoles- and echinocandin-resistant *Candida* and *Aspergillus* strains, and has been granted orphan drug status by the FDA, an intravenous BSG005 Nano Lung formulation is currently under development to further optimize targeted lung delivery^47^. Our study provides a compelling preclinical rationale for further evaluation of BSG005 in both SCFM2-based in vitro assays and the zebrafish CF infection model. On the other hand, given that azoles and echinocandins remain the cornerstone of antifungal therapy in the management of fungal infections in CF patients, future studies should prioritize the evaluation of fluconazole, voriconazole, itraconazole, and isavuconazole under CF-mimicking nutritional and gas conditions. Expanding such analyses to include a broader range of clinical CF isolates may provide more clinically relevant insights into antifungal efficacy, fungal adaptation, and therapeutic outcomes within an environment that more accurately reflects the CF lung microenvironment.

In conclusion, our findings reposition *C. albicans* as an active pathogenic contributor to CF lung disease and establish a foundation for a translational pipeline that integrates patient-derived isolates, physiologically relevant infection conditions, and host-specific models. This integrative platform provides a powerful tool for evaluating the virulence potential of a broader spectrum of clinical CF isolates, uncovering context-specific mechanisms of pathogenicity, and supporting the preclinical development of novel antifungal and anti-virulence strategies. In particular, therapeutic strategies that inhibit context-dependent morphogenesis and candidalysin-mediated epithelial damage may offer promising avenues for managing fungal complications in CF.

## Methods

### Chemicals

Unless stated otherwise, most of the chemicals used in this study, including solvents (such as DMSO), buffers (such as MOPS), polyvinylpyrrolidone (PVP), MS-222 (tricaine), antifungal drugs (including nystatin, fluconazole, and miconazole), and components of microbiological media were obtained from Sigma, Germany. HiCrome™ Candida Differential Agar was purchased from HiMedia Laboratories, India. Crystal violet and acetic acid were obtained from Serva, Germany. Nourseothricin was obtained from Werner Bioagents, Germany. Morpholinos were sourced from Gene Tools, USA. The QIAamp DNA Mini Kit was purchased from Qiagen, Germany. The GeneJET PCR Purification Kit, RapidOut DNA Removal Kit, RevertAid Reverse Transcriptase, random hexamer primer and CellTracker™ Red CMTPX were purchased from Thermo Fisher Scientific, USA. Phusion High-Fidelity DNA Polymerase, DNA ladder, restriction enzymes, T4 ligase and corresponding buffers were supplied by New England Biolabs, USA. The membranes used for Southern blot as well as the Amersham ECL™ Direct Nucleic Acid Labelling and Detection System were purchased from GE Healthcare, UK. The NucleoSpin Plasmid miniprep kit was obtained from Macherey-Nagel, Germany. Primers were purchased from Eurofins Genomics, Germany or Integrated DNA Technologies, Belgium. BigDye™ Terminator v3.1 Cycle Sequencing Kit was obtained from Applied Biosystems, USA.

### Strains and growth conditions

All strains used in this study are listed in Table S1. The *Candida albicans* reference strain SC5314 was obtained from the American Type Culture Collection (ATCC). The sputa from cystic fibrosis patients, were obtained from the Mother and Child Health Institute of Serbia and used for selective isolation of clinical fungal strains. Ethical approval was obtained from the same institution (no. 8/72). The fungal strains were stored as frozen stocks with 20% glycerol at -80 °C and sub-cultured on YPD agar plates (10 g yeast extract, 20 g peptone, 20 g glucose, 15 g agar per litre) at 30 °C. Strains were routinely grown in YPD liquid medium at 30 °C in a shaking incubator. For the selection of nourseothricin-resistant transformants, 200 µg/mL nourseothricin was added to YPD agar plates. Stock solutions of all antifungals were prepared in DMSO and stored at 4 °C prior to use.

### Isolation and identification of *C. albicans*

Fungi were isolated from sputa using selective and differential medium HiCrome™ *Candida* Differential Agar. Briefly, 1 mL of each sputum sample was mixed with 1 mL of phosphate buffered saline (PBS) (8 g NaCl, 0.2 g KCl, 0.24 g KH_2_OP_4_, 2.87 g Na_2_HPO_4_7H_2_O per litre, pH = 7.2), vortexed three times and serial dilutions were plated. After 24-hour incubation at 37 °C, the colonies of light green colour, a characteristic phenotype of *C. albicans* on this medium, were picked for further analysis.

To confirm that the collected isolates belong to *C. albicans*, the genomic DNA was extracted using the QIAamp DNA Mini Kit according to the instructions of the manufacturer. Fungi-specific ITS1 and ITS4 primers (Table S2) were used for PCR amplification of the internal transcribed spacer (ITS) region of the fungal DNA^48^. The sequence was amplified in 35 cycles with annealing temperature of 50 °C and 1 min of elongation at 72 °C. The PCR products were purified with GeneJET PCR Purification Kit, sequenced from both ends using BigDye™ Terminator v3.1 Cycle Sequencing Kit and run on Applied Biosystems® 3130 Genetic Analyzer (Applied Biosystems®, USA). Sequences were analysed and assembled with the SeqMan tool from DNASTAR Lasergene Software (DNASTAR, USA). A similarity search was performed by the BLASTn program in the NCBI GenBank database. The ITS sequences were deposited in the GenBank under accession numbers PV112556, PV112557, PV112558, PV112559, PV112560 and PV112561.

### Antimicrobial susceptibility

The antifungal susceptibility profile was assessed by determining the minimum inhibitory concentration (MIC) of the tested antifungals according to the standard broth microdilution assay, recommended by EUCAST^49^. The assay was performed in 96-well microtiter plates, employing serial twofold dilutions of the tested drugs in a final volume of 200 µL of RMPI-1640 medium with 2% of glucose, with a final inoculum concentration of 10^4^ cells/ mL. All antifungals were tested in triplicates. The MIC values were determined after 24 h of incubation at 37 °C without shaking. To assess the influence of oxygen shift on the activity of antifungal drug, treated fungal culture were incubated under ambient (21% O_2_), 5% CO_2_ and hypoxic (anaerobic jar with GasPack, Oxoid, Hampshire, UK) conditions. The lowest concentration inhibiting visible growth upon 24-hour incubation was determined to be the MIC value.

### Filamentation assay

The filamentation potential of tested isolates was examined under varying solid and liquid growth conditions that stimulate *C. albicans* filamentation. Experiments using solid media were performed using 24-well plates, while experiments in liquid media were performed in 96-well plates. Tested media included SCFM2^21^, GlcNAc^50^, Spider^51^, SLAD^52^, Lee^53^, and RPMI 1640^54^. Briefly, *C. albicans* cultures grown overnight at 30 °C on a rotary shaker (180 rpm) were washed three times with sterile PBS, diluted to a concentration of 2 × 10^6^ cells/mL, and 2 µL of this suspension was inoculated onto the surface of 1 mL solid media or into 100 µL of liquid media. Inoculated plates were incubated at 37 °C for 18 h (liquid media) or 3–5 days (solid media) and inspected daily for filamentation. Individual cells or colonies were photographed using a stereomicroscope (Carl Zeiss™ Stemi 508 doc Stereomicroscope, Germany) and a fluorescence microscope (Olympus BX51, Japan). To assess filamentous growth, colony diameter and filament length (mean from three measurements taken in three different directions), as well as hyphal density, were determined using ImageJ program. Radial filamentation was quantified by calculating the relative expansion of hyphal outgrowth from the colony centre, as previously described^27^. Filamentation pattern in SCFM2 and RPMI media was evaluated using a semi-quantitative scoring system (Figure S5) based on filament length (no filaments - 0, short - 1, moderate - 2, and long – 3; length categories were defined by visual estimation) and the growth and organization of filaments into tuft- or aggregate-like structures (no tufts – 0 and tufts formed - 1).

### Biofilm formation

The ability of tested isolates to form biofilms in liquid culture was determined using a standard 96-well microtiter plate assay (Sarstedt, Germany), with modifications^55^. Briefly, overnight cultures grown in YPD were washed three times with sterile PBS and resuspended in the appropriate test medium to a final concentration of 2 × 10⁶ CFU/mL. Each strain was tested in eight replicates. The plates were incubated for 48 h at 37 °C and 5% CO_2_.

After incubation, planktonic cells were removed, and the wells were washed twice with PBS to remove non-adherent cells. The total biofilm biomass was stained by adding 150 μL of 0.1% crystal violet solution, followed by a 20-minute incubation at room temperature. The plates were washed and dried, and the stain was solubilized by adding 150 μL of 33% acetic acid. Biofilm quantification was performed by measuring absorbance at 590 nm (OD_590_) using a Tecan Infinite 200 Pro Multiplate Reader (Tecan Group Ltd., Switzerland). Strains were categorized by the biofilm-forming capacity following the next criterion: OD_590_ > 3 (strong), 1 < OD_590_ ≤ 3 (moderate), 0.2 < OD_590_ ≤ 1 (low), OD_590_ ≤ 0.2 (non-biofilm forming).

### Strain constructions

The *ECE1* upstream region was amplified from genomic DNA of *C. albicans* SC5314 using primers ECE1.01 and ECE1.02. The *ECE1* downstream region was amplified with primers ECE1.03 and ECE1.04. Primer sequences are listed in Table S2. The PCR products were purified and digested with ApaI/SalI and PstI/SacI, respectively, and substituted for the flanking sequences in plasmid pOPT1G22^56^, resulting in pECE1G1. Plasmid pECE1M1 was generated by inserting an XhoI-SacII fragment containing the *SAT1* flipper cassette from plasmid pSFS5^57^ between the *ECE1* upstream and downstream sequences of the SalI/SacII-digested pECE1G1. Plasmids were harvested from *E. coli* via the NucleoSpin Plasmid miniprep kit (Macherey-Nagel).

The gel-purified insertions from plasmids pECE1G1 and pECE1M1 were used to transform *C. albicans* strains by electroporation^58^. The correct genomic integration of all constructs and excision of the SAT1 flipper cassette were confirmed by Southern hybridization using the flanking sequences as probes.

### Isolation of genomic DNA and Southern hybridization

Genomic DNA from *C. albicans* strains was isolated as previously described^59^. The DNA was digested with appropriate restriction enzymes, separated on a 1% agarose gel, transferred by vacuum blotting onto a nylon membrane, and fixed by UV crosslinking. Southern hybridization with enhanced chemiluminescence-labelled probes was performed with the Amersham ECLTM Direct Nucleic Acid Labelling and Detection System (GE Healthcare UK Limited, Little Chalfont Buckinghamshire, UK) according to the manufacturer’s instructions.

### in vivo experiments in the zebrafish (*Danio rerio*) model

All experiments with zebrafish embryos were performed in accordance with the European Directive 2010/63/EU and the Ethical Guidelines for the Care and Use of Laboratory Animals of the Institute of Molecular Genetics and Genetic Engineering of the University of Belgrade. Wild-type (AB) zebrafish embryos, kindly provided by Dr. Ana Cvejić (Wellcome Trust Sanger Institute, Cambridge, UK), were raised to the adult stage in a temperature-and light-controlled zebrafish facility at 28 °C and a standard 14:10 hour light-dark photoperiod. The fish were fed twice daily with commercial dry food (SDS200 and SDS300 granular food; Special Diet Services, Essex; UK and TetraMinTM flakes; TetraMelle, Germany) and daily with *Artemia nauplii*.

### Morpholino injection

Splice-blocking morpholino targeting cftr (5’-GACACATTTTGGACACTCACACCAA-3’) was injected into one-cell-stage zebrafish embryos (Figure S6) according to the previously described procedure^43^. The efficiency of gene knockdown was confirmed by RT-PCR. Briefly, the RNA was isolated from zebrafish embryos at 48 h post-fertilisation (hpf) or 120 hpf, following previously described procedure^60^. The RNA pellet was resuspended in 50 µL of nuclease-free water and residual DNA was removed using the RapidOut DNA Removal Kit, following the manufacturer’s instructions. RevertAid Reverse Transcriptase was used to synthesize first-strand cDNA with random hexamer primer from 1 μg of total RNA at 50 °C for 50 min. The efficiency of gene knockdown was confirmed by RT-PCR using CFTR-F and CFTR-R primers (Table S2), complementary to both sides of the MO sequence. A standard control-MO (5’-CCTCTTACCTCAGTTACAATTTATA-3’) was included as a negative control in all experiments.

### Zebrafish model of lethal disseminated candidiasis

All experiments involving zebrafish embryos were performed in compliance with the European directive 2010/63/EU and the ethical guidelines for the care and use of laboratory animals stipulated by the Institute of Molecular Genetics and Genetic Engineering, University of Belgrade. Wild type (AB) zebrafish were raised to adult stage in a temperature- and light-controlled zebrafish facility at 28 °C and a standard 14:10-hour light-dark photoperiod, fed with commercial dry food (SDS300 granular food; Special Diet Services, Essex; UK and TetraMinTM flakes; Tetra Melle, Germany) twice a day, and with *Artemia nauplii* daily. Embryos produced by pair-wise mating were collected, washed to remove debris, distributed at 10 embryos per well into 24-well plates containing 1 mL E3 medium (0.292 g NaCl, 0.0126 g KCl, 0.048 g CaCl_2_ x 2H_2_O and 0.082 g MgSO_4_ x 7H_2_O per litre), and kept at 28 °C.

#### *C. albicans* culture and preparation of the cells for microinjection

The fungal cultures grown overnight in YPD broth at rotary shaker (180 rpm) at 30 °C were sub-cultured at a ratio of 1:100 under the same growth conditions to reach a mid-exponential phase (OD_530nm_ 0.7-0.8). Unless using reporter strains, cells were labelled using CellTracker™ Red CMTPX (2 µM final concentration), according to the manufacturer’s instructions. After centrifugation at 2200 × *g* for 5 min (Centrifuge 5415D, Eppendorf, Hamburg, Germany) and washing three times with sterile 1×PBS, the fungal cells were resuspended in 2% PVP to a final concentration of 2×10^7^ cells/mL.

#### Infection of zebrafish embryos

Prior to each experiment, zebrafish embryos (both CFTR⁺ and CFTR⁻) were manually dechorionated at 24 h post-fertilisation (hpf). At 34 hpf, dechorionated embryos were anesthetized using tricaine (200 µg/mL) and microinjected with ~5 nL of fungal cell suspension into the hindbrain via the otic vesicle, delivering a dose of 55-75 fungal cells per embryo. Following injection, embryos were allowed to recover for 1 h at 28 °C, after which they were distributed into a 24-well plate (10 embryos per well) and incubated at 28 °C. Embryos injected with 2% PVP alone served as the mock-infected control group. For each fungal strain, 20 embryos were used per experimental replicate, and the experiment was performed three independent times (n = 60 embryos per strain).

#### Treatment of infected embryos

*C. albicans*-infected CFTR+ and CFTR- embryos were exposed to 1 µM of polyene drug nystatin, the highest non-toxic drug concentration selected according to our previous studies^41,42^. Total of 20 embryos per treatment were used; the experiment was repeated three times (n = 60). Treated embryos were incubated at 28 °C and the antifungal effect of nystatin was assessed daily for 4 days post infection (dpi) inspecting *C. albicans* growth and filamentation, as well as the survival of infected embryos compared to the control (untreated infected) embryos.

### Statistical analysis

Differences in filament length and hyphal density between media were assessed by one-way ANOVA followed by Bonferroni post-hoc test. The association between radial filamentation and hyphal density across different media was assessed using Spearman’s rank correlation coefficient (ρ) to account for potential non-linear relationships and non-normal distribution. Linear regression lines were plotted for visual illustration of trends across media, but were not used for statistical inference. Evaluation of the effect of gas composition on the growth of *C. albicans* isolates in SCFM2 medium was performed by comparing the optical density values using the non-parametric Friedman test for repeated measures. Post hoc pairwise comparisons were performed using the Wilcoxon signed-rank test with Bonferroni correction to adjust for multiple comparisons. Fold changes between gas conditions were calculated to quantify the relative differences in growth across conditions. The difference in the proportion of embryos with or without hyphae between CFTR+ and CFTR- embryo groups was analysed using the χ² test, while the differences in fungal burden, assessed by fluorescence intensity (measured in pixels), were analysed using one-way ANOVA followed by Bonferroni post hoc test. Survival analysis was performed using the Kaplan–Meier method, and comparisons between survival curves were conducted using the log-rank test. All statistical analyses were carried out using Phyton or GraphPad Prism version 9.0 (GraphPad Software, San Diego, CA, USA).

## Supplementary information

Radakovic et al_Supplementary Information

## Data Availability

All data supporting the findings of this study are included in the article and its supplementary files and figures. All additional data are available from the corresponding authors upon request. ITS sequence data supporting CF isolates identification have been deposited in the NCBI GenBank under accession numbers PV112556-PV112561.
