# Supplementary material for: Beyond colonization: Candida albicans exhibits substantial pathogenic potential in cystic fibrosis environments
Source: NPJ Biofilms Microbiomes. 2025 Dec 19;12:23. doi: 10.1038/s41522-025-00889-2 (PMC12848021; doi:10.1038/s41522-025-00889-2)
Supplement: Supplementary file 1 — Radakovic et al_Supplementary Information [file 41522_2025_889_MOESM1_ESM.pdf]

Beyond colonization: *Candida albicans* exhibits substantial pathogenic potential in cystic fibrosis environments

Natasa Radakovic[1], Nikola Plackic[1], Jelena Djuris[2], Joachim Morschhäuser[3], Aleksandar Sovtic[4,5], Mihail Basa[4], Predrag Minic[4], Fabio Zobi[6], Aleksandar Pavic[1]*

[1]Institute of Molecular Genetics and Genetic Engineering, University of Belgrade, Vojvode Stepe 444a, 11042 Belgrade, Serbia

[2]University of Belgrade - Faculty of Pharmacy, Vojvode Stepe 450, 11221 Belgrade, the Republic of Serbia

[3]Institute of Molecular Infection Biology, University of Würzburg, Würzburg, Germany

[4] Mother and Child Health Institute of Serbia "Dr. Vukan Čupić"

[5]Faculty of Medicine, University of Belgrade, Serbia

[6]Department of Chemistry, Fribourg University, Chemin du Musée 9, 1700 Fribourg, Switzerland

Content:

Table S1. List of *C. albicans* strains used in this study

Table S2. List of primers used in this study

Figure S1. Comparative analysis of biofilm formed by CF clinical isolates in SCFM2 in relation to other media

Figure S2. Correlation analysis plotting between biofilm and radial filamentation or hyphal density

Figure S3. The influence of different gaseous conditions on growth of CF isolates and the SC5314 reference strain in SCFM2

Figure S4. Filamentation potential of clinical CF isolates of *C. albicans* influenced by gas composition (21% O_2, 5% CO_2 and hypoxia) and nutrient environment (SCFM2 vs. RPMI)

Figure S5. Dose-dependent effect of nystatin on filamentation of clinical CF isolates and the SC5314 reference strain upon three different gaseous conditions in SCFM2 and RPMI

Figure S6. Generation of CFTR zebrafish morphants using the splice-targeting morpholino

Figure S7. CFTR-deficient zebrafish embryos reveal enhanced *Candida albicans* virulence, increased *ECE1* expression, and decreased therapeutic efficacy of nystatin *in vivo* (colour adjusted version of Fig. 5)

Table S1. List of *Candida albicans* strains used in this study

Strain	Parental strain	Characteristics/ Genotype	Reference
SC5314		Wild-type reference strain	Gillum et al.
CF1a		Clinical isolate from CF sputum	This study
CF3a		Clinical isolate from CF sputum	This study
CF5a		Clinical isolate from CF sputum	This study
CF5b		Clinical isolate from CF sputum	This study
CF7a		Clinical isolate from CF sputum	This study
CF8a		Clinical isolate from CF sputum	This study
SCECE1G1	SC5314	*ECE1*/*ece1*::P*ECE1-GFP-caSAT1*	This study
P1ECE1G1	CF1a	*ECE1*/*ece1*::P*ECE1-GFP-caSAT1*	This study
P5ECE1G1	CF5a	*ECE1*/*ece1*::P*ECE1-GFP-caSAT1*	This study
SCECE1M4	SC5314	*ece1*Δ::*FRT*/*ece1*Δ::*FRT*	This study
P1ECE1M4	CF1a	*ece1*Δ::*FRT*/*ece1*Δ::*FRT*	This study
P5ECE1M4	CF5a	*ece1*Δ::*FRT*/*ece1*Δ::*FRT*	This study

Gillum AM, Tsay EY, Kirsch DR. Isolation of the *Candida albicans* gene for orotidine-5′-phosphate decarboxylase by complementation of *S. cerevisiae ura3* and *E. coli pyrF* mutations. Molecular and General Genetics MGG. 1984;198(1):179-82.

Table S2. List of the primers used in this study

Designation	Sequence (restriction sites are underlined)	Reference
ITS-1	5'-TCCGTAGGTGAACCTGCGG-3'	White *et al*, 1990.
ITS-4	5'-TCCTCCGCTTATTGATATGC-3'	White *et al*, 1990.
ECE.01	5'-ATATGGGCCCATTTGTAGGATTTTCAGCAG-3' (*Apa*I)	This study
ECE.02	5'-ATATGTCGACGAATGGAAAATAGTTGGTAG-3' (*Sal*I)	This study
ECE.03	5'-ATATCTGCAGCCGCGGCTCAGCAGATAAAAATTTGTTTTCC-3' (*PstI, SacII*)	This study
ECE.04	5'-ATATGAGCTCTTGTTAGGTTGCCATAAACAG-3' (*SacI*)	This study
EEF1α_F	5'-TCTGTTACCTGGCAAAGGG-3'	Bernut *et al*, 2016
EEF1α_R	5'-TTCAGTTTGTCCAACACCCA-3'	Bernut *et al*, 2016
CFTR-F	5'-CCTGTGGAGGATGCCAACTGCC-3'	Bernut *et al*, 2019
CFTR-R	5'-TGCATGCCCAGGTGGTGCAG-3'	Bernut *et al*, 2019

White T, Bruns T, Lee S, TaylorJ. Amplification and direct sequencing of fungal ribosomal RNA genes for phylogenetics.PCR-protocols a guide to methods and applications. Edited by: InnisMA, Gelfand DH, Sninski JJ, White TJ. 1990, SanDiego: Academic press, 315-322.

Bernut, A, Nguyen-Chi, M., Halloum, I, Herrmann, J. L, Lutfalla, G, Kremer, L. Mycobacterium abscessus-induced granuloma formation is strictly dependent on TNF signaling and neutrophil trafficking. PLoS pathogens 2016; 12(11), e1005986.

Bernut, A, Dupont, C, Ogryzko, N. V, Neyret, A, Herrmann, J. L et al. CFTR protects against Mycobacterium abscessus infection by fine-tuning host oxidative defenses. Cell reports 2019; 26(7), 1828-1840.

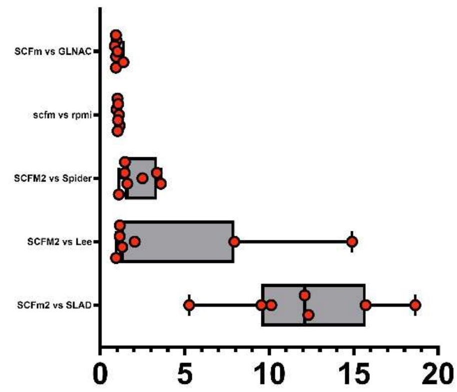

Figure S1. Comparative analysis of biofilm formed by clinical CF isolates in SCFM2 in relation to other filamentation-inducing media, indicating that SCFM2 supports more robust biofilms compared to SLAD, Lee and Spider media.

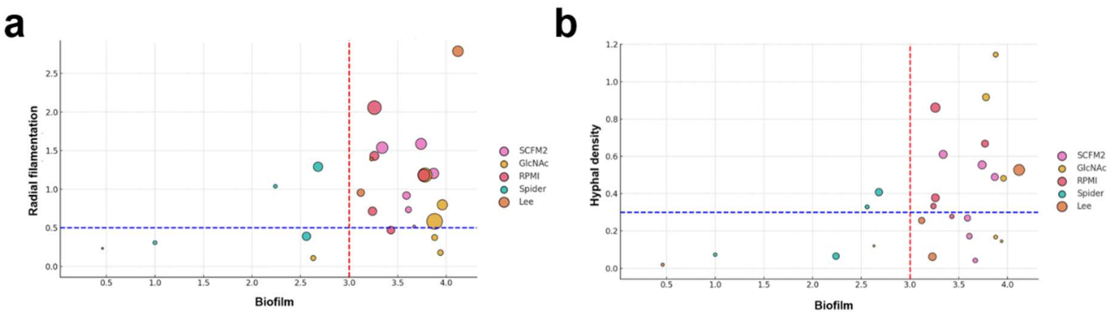

Figure S2. Correlation analysis plotting between biofilm and a) radial filamentation or b) hyphal density, assessed for clinical CF isolates across each of six different media including SCFM2, Spider, RPMI, GlcNAc, SLAD and Lee media.

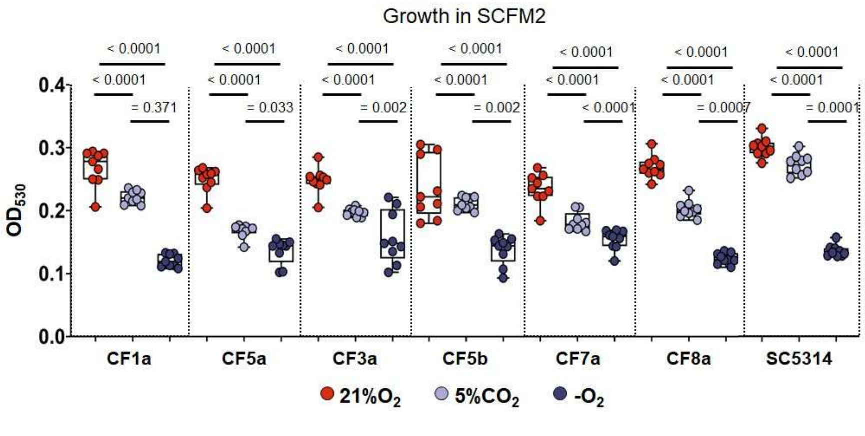

Figure S3. The influence of different gaseous conditions on growth of CF isolates and the SC5314 reference strain in SCFM2 (CF-mimicking conditions). One-way ANOVA with the Bonferroni test was used to determine statistical significance between groups.

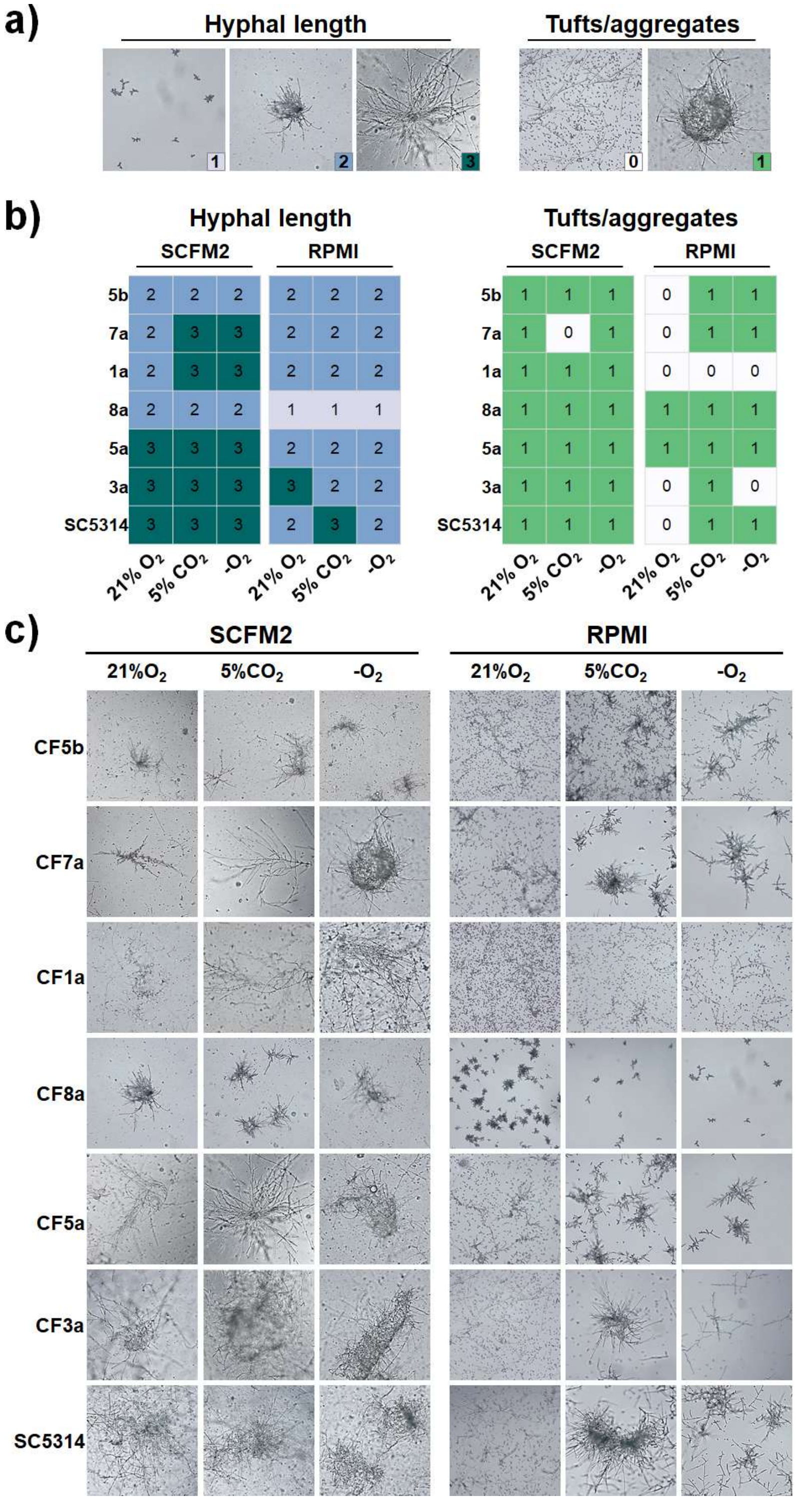

Figure S4. Filamentation potential of clinical CF isolates of *C. albicans* influenced by gas composition (21% O_2, 5% CO_2 and hypoxia) and nutrient environment (SCFM2 vs. RPMI). a) Semi-quantitative scoring of filamentation based on a) the filamentation size (no filaments - 0, short - 1, moderate - 2, and long - 3) and b) the growth and organization of filaments into tuft- or aggregate-like structures (no tufts - 0, and tufts formed - 1). c) Representative images of filamentation phenotype of clinical CF isolates upon different growth conditions are shown. The reference strain SC5314 was used as control.

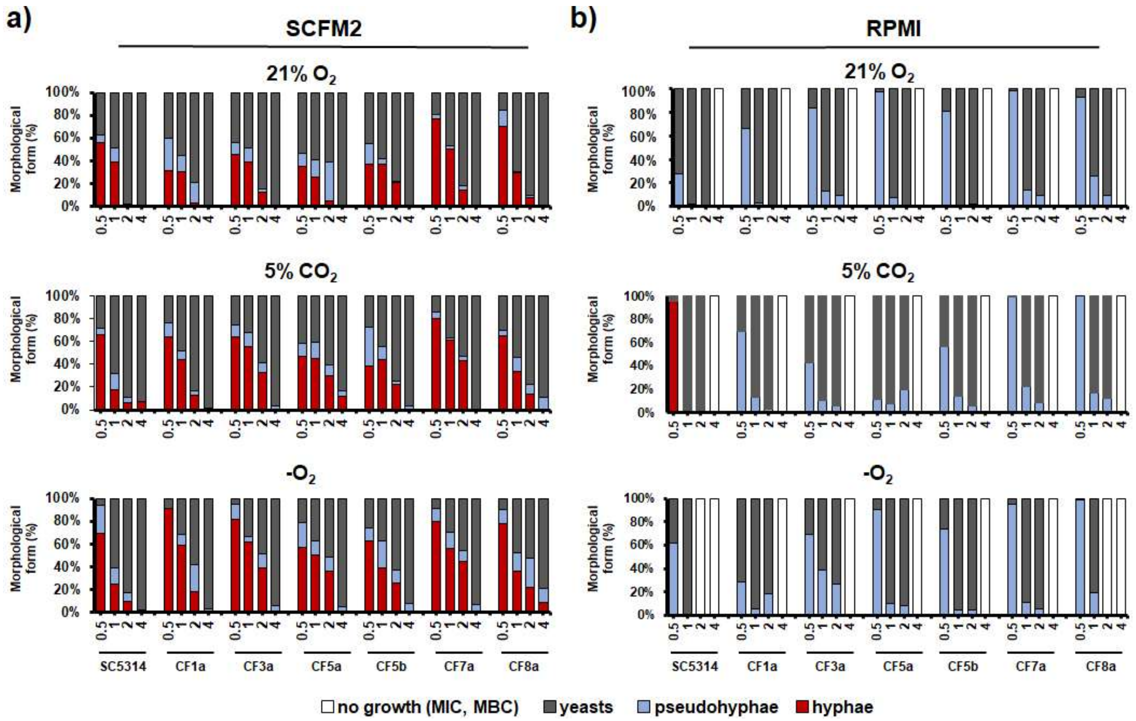

Figure S5. Dose-dependent effect of nystatin on filamentation of clinical CF isolates and the SC5314 reference strain upon three different gaseous conditions (21% O_2, 5% CO_2 and hypoxic) in a) SCFM2 and b) RPMI medium.

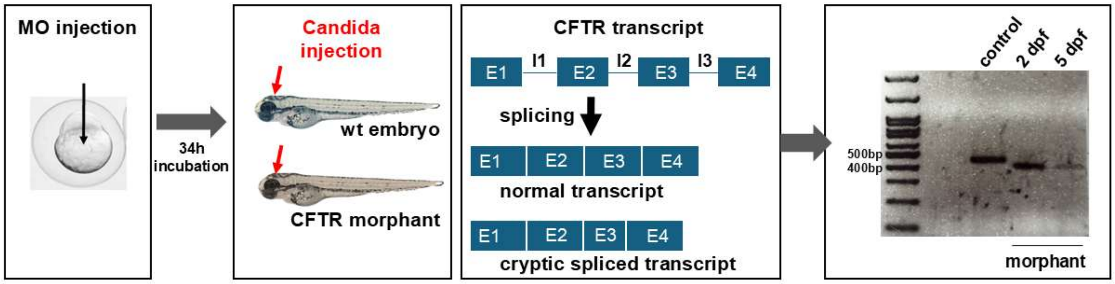

Figure S6. Generation of CFTR zebrafish morphants using the splice-targeting morpholino. A schematic representation. A splicing-targeting morpholino was injected in the 1-cell stage embryos. At the 34 hours post-fertilisation (hpf) stadium, the embryos were injected with 55-75 cells of different *C. albicans* strains and their survival was tracked until 5 days post-fertilisation (dpf). RT-PCR followed by agarose gel electrophoresis confirmed shorter fragments of alternative spliced transcripts in CFTR morphants.

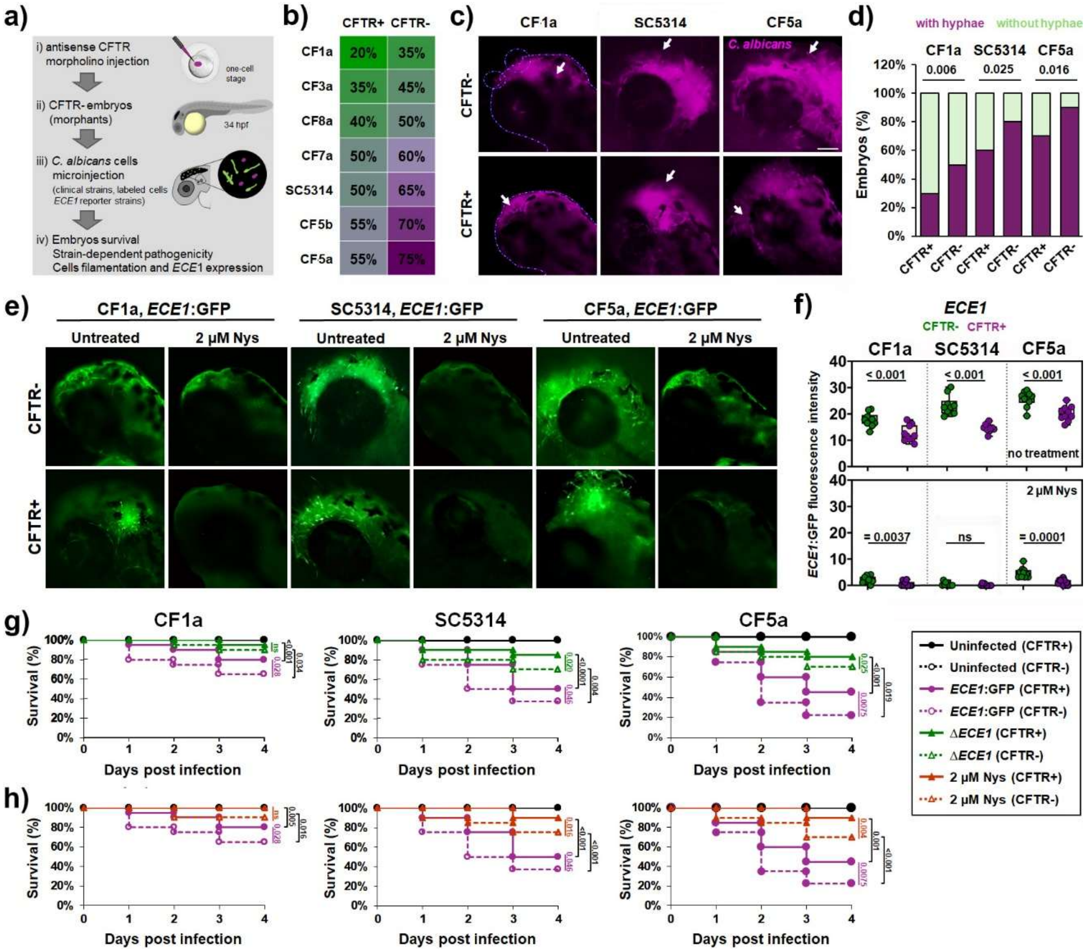

Figure 7. CFTR-deficient zebrafish embryos reveal enhanced *Candida albicans* virulence, increased *ECE1* expression, and decreased therapeutic efficacy of nystatin *in vivo*. a) Schematic overview of the newly developed *C. albicans* infection model using CFTR-deficient (CFTR$^-$) embryos, generated via CFTR knockdown, to mimic the CF host environment. **b)** Colour-coded map showing the survival rates of CFTR$^+$ and CFTR$^-$ embryos (n = 20) at 120 hours post-fertilisation (hpf), following infection with *C. albicans* isolates derived from the sputum of CF patients. The fluorescence colour of RFP-expressing *C. albicans* was modified for enhanced visibility and to accommodate colour-blind accessibility. **c)** Representative fluorescence images of infected embryos showing extensive hyphal growth (arrow) within tissues and epithelial barrier breaching in the head region (dashed outline). The SC5314 reference strain was used as control. Fungal cells were labelled with a red cell tracker. The colour was subsequently adjusted for colour-blind accessibility. **d)** Proportion of CFTR$^-$ versus CFTR$^+$ embryos with or without visible hyphae in the hindbrain at 24 hours post-infection (hpi), assessed by fluorescence microscopy (n = 20). Statistical significance was evaluated using the $\chi 2$ test by comparing the distribution of embryos with and without hyphae between CFTR$^+$ and CFTR$^-$ groups. **e)** *In vivo* fluorescence of *ECE1:*GFP reporter strains upon infection of CFTR$^-$ and CFTR$^+$ zebrafish embryos, with or without nystatin treatment. **f)** Quantification of GFP fluorescence intensity as a measure of *ECE1* expression under the same conditions. **g)** Contribution of *ECE1* to *C. albicans* pathogenicity assessed by Kaplan–Meier survival analysis of CFTR$^-$ and CFTR$^+$ embryos infected with either *ECE1*:GFP reporter strains or *ECE1* deletion mutants. **h)** Validation of the newly established infection model by assessing the antifungal efficacy of nystatin at 2 µM. Survival differences between CFTR$^+$ and CFTR$^-$ embryos under each condition were statistically analysed using the log-rank (Mantel–Cox) test. Data for the *ECE1:GFP* strain are identical in panels g and h, as both analyses were performed within the same experiment. In panel g, *ECE1:GFP* serves as a reference strain for the deletion mutants, while in panel h it represents the untreated control for the nystatin-treated groups.
